# Near-Field Communication (NFC) Cyber Threats and Mitigation Solutions in Payment Transactions: A Review

**DOI:** 10.3390/s24237423

**Published:** 2024-11-21

**Authors:** Princewill Onumadu, Hossein Abroshan

**Affiliations:** School of Computing and Information Sciences, Anglia Ruskin University, Cambridge CB1 1PT, UK

**Keywords:** NFC, NFC-enabled devices, digital money transactions, RFID, EMV cards, cyber risks, digital privacy, contactless payment regulations, cyber security

## Abstract

Today, many businesses use near-field communications (NFC) payment solutions, which allow them to receive payments from customers quickly and smoothly. However, this technology comes with cyber security risks which must be analyzed and mitigated. This study explores the cyber risks associated with NFC transactions and examines strategies for mitigating these risks, focusing on payment devices. This paper provides an overview of NFC technology, related security vulnerabilities, privacy concerns, and fraudulent activities. It then investigates payment devices such as smartphones, contactless cards, and wearables, highlighting their features and vulnerabilities. The study also examines encryption, authentication, tokenization, biometric authentication, and fraud detection methods as risk mitigation strategies. The paper synthesizes theoretical frameworks to provide insights into NFC transaction security and offers stakeholder recommendations.

## 1. Introduction

Digital technology has brought about significant changes in how businesses operate globally, particularly in purchasing items and paying for transactions [1,2]. In the past few years, how businesses are performed globally has shifted from the ordinary (less technology-based) to the advanced (more technology-based and dependent) form with the aid of advanced technological devices enabling established financial communications. Within the class of these advanced technological devices, near-field communication (NFC) is a top-class technology that is still under study [3]. NFC is an emerging digital technology used in payments and transaction settlements. NFC, as the name implies, is a technology that enables short-range communications using the ideas of Radio Frequency Identification (RFID) [4] to facilitate and activate communication between the mobile devices of billions of people all over the world [5,6]. NFC, as a credible technological advancement, can be extremely reliable in everyday transactions, with enabling functionalities that allow ease in making quick and convenient contactless payments using smartphones, smartwatches, and other devices [7].

Although different wireless technologies, like Bluetooth, Wi-Fi, infrared, and others, have technical and functional differences, there are similarities, like data sharing between connected devices. NFC also provides some level of efficiency in sharing information between interconnected devices at close range [8,9,10]. NFC technology is an advancement from Radio Frequency Identification Technology (RFID), which is used in security scan cards and other online payment mediums [11]. Although several types of wireless and contactless technologies exist, mobile and payment service providers mainly use NFC technology as it is engineered to function over extremely short distances, typically less than 4 cm, and is seen as a powerful tool for addressing emerging customer demands and enabling value-added business models [12].

NFC payments occur when a Europay, MasterCard, or Visa (EMV) card communicates with a payment terminal, sending encrypted payment information from the customer to the retailer using smart devices such as smartphones and smartwatches or tap-to-pay cards such as EMV credit/debit cards [13,14,15]. Despite the significance of this technology in today’s business affairs, many potential risks lurk around the use of this technology while making payments for transactions, birthing the need for this study research [16,17]. Arguably, NFC-enabled devices leave us with many attendant risks of losing both financial and personal data/assets. These attendant risks include eavesdropping, data corruption, payment processing fraud, mobile malware attacks, theft, card cloning, interception attacks, and consumer privacy risks [18,19].

In past literature, these payment options and their associated cyber risks distorting a seamless payment procedure have been explored. However, more studies and mitigation solutions are still needed to reduce payment risks that occur while using digital payment technologies. Accordingly, this study will conduct a comprehensive and systematic literature review of past research works in NFC-enabled payment options to unravel the attendant risks and find the best ways to mitigate the future occurrence of these risks. Significantly, this study benefits businesses, smart device users, NFC-related solution developers, payment service providers, and other fintech industries and will serve as a basis for further research. Table 1 analyses some of the previous surveys on this topic, highlighting their focuses, contributions, and shortcomings.

This study is an in-depth systematic literature review (SLR) aimed at exploring the past works of other researchers regarding NFC transactions. It aims to identify the shadow points not covered by previous research and propose solutions to these shadow points. An in-depth study will also be conducted into risks affecting NFC transactions and how to limit their occurrences in future time zones.

The SLR shall include an extensive study of topics and subject matters from sourced academic databases, journals, and industry reports to gather information on the various types of NFC-enabled devices that can be used for payment at transaction points, potential associated risks, steps to mitigate the associated risks and the importance of reducing these risks. The SLR will only include studies from 2017 to the present. This study uses an inductive qualitative research approach to widely collect the necessary data to provide sufficient answers to the research questions, focusing on using the Saunders research onion model [23].

The rest of the paper is organized as follows: The study’s methodology is described in the next section. The results of the study’s reviews, such as NFC technology, risks, and mitigation solutions, are explained in the second section. The last two sections discuss the findings, the study’s limitations and future works, and the paper’s conclusion.

## 2. Methodology

The results analyzed for this study are based on secondary data collected from past research work. To effectively understand the topic of discussion, this study conducted an in-depth SLR, which allowed us to select several pieces of literature published on the subject matter “NFC technology” for in-depth review and analysis. From this, our data are drawn, and the research work is based.

To provide strong research results, our search process was based on keywords that aligned with our topic of discussion. Most of the keywords used are:

Keyword 1: “NFC” && “Risks”.
Keyword 2: “NFC devices” && “Security”.
Keyword 3: “NFC” && “Privacy”.
Keyword 4: “NFC” && “Technology”.

The database used to achieve the SLR objective was gathered and adopted from various research sites and platforms on SpringerLink, ACM Library, Conestoga Library, Google Scholar, Scribbr Link, Staxpayments, Researchgates, ProQuest Central, Gale General OneFile, ABI/INFORM Trade & Industry, Gale OneFile, Nexis Uni, EBSCOhost, EBSCOhost Academic Search Complete, PressReader, Advanced Technologies & Aerospace Collection, EBSCOhost Business Source Complete, and Regional Business News. The results found from journals, magazines, newspaper articles, dissertations, text resources, conference proceedings, government sources, archive notes, symposiums, articles/conferences, and articles/book chapters were classified based on some specific exclusion and inclusion criteria, and these will be presented in detail the following subheadings.

To effectively achieve the study’s research objectives, extracted data were collected and classified based on the following exclusion and inclusion criteria, which are mentioned below.

The results found from the above sources for the publications dated 2017–2024 were analyzed in this study. In total, 6348 publications and websites were selected to be investigated in this SLR:

- Another metric we considered was the use of literature written in English, and we obtained these results after the first extraction. Due to the use of English criteria, the study materials used were reduced from 6348 to 5563.
- The 5563 studies were double-checked to avoid repeated use of study materials.

**Figure 1:**
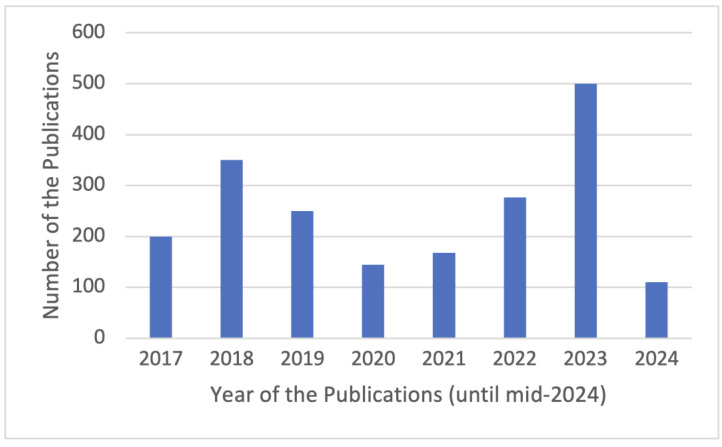
Number of selected studies by year.

**Figure 2:**
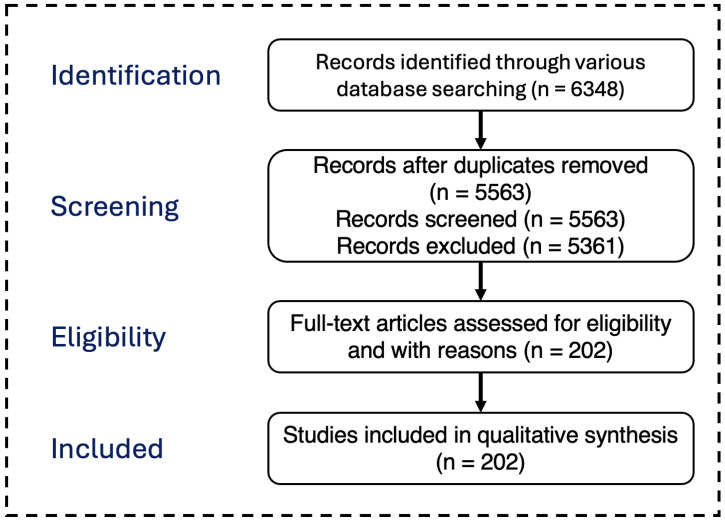
Schematic diagram PRISMA Literature Review.

Moreover, to conveniently control and organize the study items collected, materials were extracted based on title, abstract, scope of research, and the important ideology behind our selection. Figure 1 shows the number of selected publications per year from 2017 to mid-2024. We finally determined whether the realized study had a strong link with our basic insight. We considered studies that were majorly focused on NFC and RFID; thus, 202 publications were selected and extracted for the analysis process. Figure 2 illustrates a diagram of the PRISMA [24] in the literature review.

## 3. NFC Technology, Risks, and Mitigation Solutions

### 3.1. NFC Technology

NFC is a “wireless personal area network (PAN) technology that connects two compatible devices in very close range to each other in order to enable slow but reliable data transfer” [25]. It is a close-range, low-frequency wireless communication technology that allows communication over NFC-compatible devices via transmitting information signals [26,27,28]. NFC technology can either be passive, such as tags and other small transmission enablers that are capable of sending information to other NFC devices without the use of a power source of their own, or active, such as smartphones and card readers that can send and receive information simultaneously, implying that the device is embedded with such features to receive and send information [27,29].

NFC uses Radio Frequency Identification (RFID) technology to send information over radio waves, like other wireless technologies, e.g., Bluetooth and Wi-Fi [30]. This technology can be very valuable; however, to operate and communicate effectively, NFC devices should comply with precise specifications and standards [31]. NFC technology operates based on older technology, which is RFID-enabled and uses electromagnetic spectrum to transmit information [27,32].

The NFC technology communicates using three unique modes of operation to determine the type of information to be exchanged. The first mode of operation is using smartphones, the most common transmission mode, via a peer-to-peer mode. Information is exchanged between two devices, allowing them to communicate in active and/or passive modes through a network [21,33]. The second mode of operation is the read/write mode, which is a one-way data transmission [21,34]. The third mode of operation is card emulation, which involves using smart or contactless credit cards to make payments for commodities or services while using public transport systems (rail, road, or sea), restaurants, and more [21,35].

### 3.2. NFC Transactions

NFC transactions only occur over the use of NFC-enabled devices, allowing customers to make simple, contactless, and convenient transactions. The NFC-enabled payment model is used for peer-to-peer (P2P) payments and peer-to-merchant (P2M) [36]. Interestingly, NFC transactions work uniquely, through which customers can log into their devices and tap them on an NFC-enabled reader to make payments [37].

Objectively, NFC’s unique payment process is dynamically designed to store information on tiny microchips or tags and transmits this information at 125/134 kHz to readers within a limited physical range [38]. Giese, Liu [38] added that transactions are done with cards that use low-frequency tags, which look like magnetic stripe cards known as “magstripes” and have no additional cryptographic protection. However, using high-frequency tags that transmit information at 13.56 MHz offers different transaction security [35]. The tags only possess a UID that is similar in security architecture to the magstripe and can be protected cryptographically due to the volatility of its security framework [38,39].

More like it is the use of NFC card payments, which has an NFC-enabled chip embedded in the physical component of the EMV credit cards (like an ATM card), to provide a fast and contactless card transaction medium [38]. The security components of these cards provide considerable security resistance to any external body, giving users confidence [40]. Additionally, NFC payments can be made using smartphones with NFC chips embedded [41]. In a quick overview, it is crucial to state that NFC transactions can only occur over NFC-enabled devices, posing many difficulties for non-enabled devices, leading to the need to either use an NFC device or update devices to include NFC features [42].

Payments over NFC-enabled devices are much more secure than payments over magnetic stripe cards [43]. Also, NFC technology is included in other forms of payment arrangements, such as mobile wallets [44]. The mobile wallet is an easy-to-use domain that uses the NFC chip installed in smartphones, which enables payment for services, ticketing, access control, and other transactions activated by users [45]. The effectiveness of NFC technology is entirely dependent on its functionality on mobile devices, NFC tags, and NFC readers [36]. NFC technology can be used in payment, health care, public transportation, retail industries, tourism, indoor navigation systems, education, automotive areas, and other significant areas not mentioned within this study research [11]. In fact, the key stakeholders determine the effectiveness of any NFC technology in performing their transactions.

### 3.3. Key Players and Stakeholders Involved in NFC Transactions

A stakeholder is any individual or group interested in any organization or the implementation of a development [46]. NFC stakeholders believe contactless payment is necessary and viable in virtually all economic sectors, coupled with advancements in innovation and technologies [47]. Stakeholders’ interest in the proceeds and activities of any organization helps to finetune the organization and its technological development. In the case of NFC-enabled devices, stakeholders are seriously interested in a technology that offers seamless processes and secured architectures while performing transactions [48].

Stakeholders have incurred quite an inestimable sum on building up NFC technology that best provides secured payment options to users, enforcing the need for customers to adopt NFC mobile wallet payment [45]. Generally, stakeholders can be internal or external and are classified further into shareholders, employees, government, and users of an organization’s services or products; this classification is like the NFC technology framework [38]. Addressing NFC’s key challenges or limitations will be done through stakeholders’ contributions toward organizational restructuring and operational transformation to build a formidable payment platform whose security framework is impenetrable or unbeatable [38]. Stakeholders in NFC transactions can be identified as:

**Users** are considered owners of NFC-enabled devices and cards (i.e., cardholders) for payment or purchase purposes. Their interest focuses on accurately designing a technology and service quality that meets all their requirements of accessing, performing, and approving transactions over secured NFC-enabled devices [49,50]. They hold a significantly high interest in ensuring the safety of their information and further financial data in cases of misplaced devices. Hence, they are to be greatly considered in the future development of NFC technology. NFC users are generally classified into terminal users and regular customers [50,51,52]. Terminal owners are usually concerned with ensuring that all devices purchased comply with minimum specifications for contactless payment terminals and devices, and they have the responsibility to identify, document, and treat risks associated with contactless payments possibly encountered while being used [51]. As a further explanation, the part two end users, who are the customers, are responsible for due diligence and the option to initiate and authorize a contactless payment over an NFC platform [51]. This explains that customers are liable to gain adequate knowledge about the full details of all transactions before executing them over the NFC platforms.

**Merchants** are receivers of any payment process while using the NFC technology [38]. As a stakeholder, the merchant is interested in ensuring seamless and secured transactions over NFC platforms, where the merchants exercise some authority to accept and validate transaction details while having sensitive information effortlessly protected. Merchants are responsible for any device issued into the market to adequately possess all features of easily making contactless transactions [51].

Merchants must ensure that a contactless symbol is displayed when users make any contactless transaction and that all records of contactless payments, data, and documents made over devices are kept safely within a particular time frame [51]. In this circular, he added that merchants should ensure customers’ authorization for certain transactions above the stated transaction limit per day and that merchants should be held responsible for any possible fraud that may occur due to their negligence or involvement.

**Device Vendors/Makers:** are responsible for the design of the devices incorporated with NFC technology [38]. These devices include smartphones, tablets, and laptops like Apple, Samsung, and others [53]. The stakeholders are interested in installing and activating NFC on devices, making the device NFC compatible. Not only will the device be compatible, but the interest of the stakeholders in this regard will be based on ensuring users’ sensitive information is adequately secured if this technology is installed on devices. The device maker is more concerned about the ability to activate or authenticate user-initiated transactions through the device design.

**Card Issuers:** includes the creator of a payment card used by end users [38]. The card issuer is interested in ensuring a seamless validation process of user-initiated transactions over the use of NFC-enabled cards while ensuring the maximum security of user-initiated transactions [41].

All these stakeholders have strong interests in the seamless operation and security of the NFC technology, birthing their relentless efforts to ensure that the NFC devices and cards are well designed to eliminate all forms of security threats [35]. Despite the relentless efforts of shareholders to provide resources and structures that provide a secure payment platform, this NFC transaction process is still volatile and exposed to risks at the time of the transactions [54] because it requires little or no additional requirements from end users [38]. Also, the volatility of the NFC framework occurs due to the incorporation of RFID in its technological build, leaving the NFC technology exposed to attacks launched over the RFID network [55].

## 4. NFC Cyber Risks

Due to technological improvements and the greater dependencies of people and organizations on information technology (IT), cybercrime has also increased, making it extremely important to take active steps to prevent the loss of personal and financial data using these new technologies [56]. Understanding the risks of individuals’ personal and financial information saved on NFC-embedded devices and the estimation that around 6.1 billion NFC-enabled handsets will be available by 2030, it becomes necessary to take steps to reduce the occurrence of these risks [57,58].

These days, businesses have moved beyond accepting physical means of payment for goods sold and services rendered. Nigeria is a solid example of this, having experienced a mobile money revolution and the activation of digital money from 2019 to the present [59].

Smart devices are either smartphones (like iPhones and Androids), laptops, tablets, or wearable devices (i-watches and other digital watches) that are NFC-enabled [60,61]. These devices are the most effortlessly and widely used devices for transaction payments, for instance, for Google Pay (which runs on Androids and tablets) and/or Apple Pay (which runs on i-devices). Individuals connect their financial details via bank ATM cards to these NFC-enabled applications to make payments or purchases on websites and online pages. Once this process is initiated, individual data are stored on the applications’ mobile wallets with access control systems like biometrics, face IDs, key locks, encrypted passwords, and motion detectors to protect individuals’ payment data [62,63]. Despite these many security layers, NFC-enabled smart devices are still much more vulnerable to several transaction risks [64]. These transaction risks include:

**Data Tampering** involves third-party interference to alter payment data while a transaction is in progress due to the improper use of access control systems [65]. It can take the form of data corruption by inundating the communication channels with incorrect information that disrupts the original message from being rightly interpreted [66].

**Smart Device Malware** is used by criminal intent persons to infect smart devices. This is carried out by manipulating NFC tags to override existing tags. It is usually placed in the payload awaiting to be scanned by an NFC-enabled device; once the scanning process is executed, the malware is downloaded and installed and then steals private, financial, and login data, granting attackers unauthorized access to people’s data [67,68].

**Eavesdropping:** Data on an NFC-enabled device can be intercepted at a distance by an attacker who listens to an NFC communication that transmits personal information [67,68,69]. This extracted and saved information by attackers can be used to commit multiple crimes, leaving the owner of those personal details culpable for those crimes or exposed to losing a fortune via these mediums [69].

Tap-to-pay cards are specially designed cards with a Wi-Fi symbol on the side and embedded NFC functionalities that allow the activation of a payment for a service through a swipe on an NFC-enabled device [21]. These types of cards are contactless and can be used at cafeterias, train stations, bus stations, and other business areas. However, they are at risk of card skimming and cloning.

**Card Skimming:** Card skimming occurs when an NFC tag can be placed at a point-of-sale terminal without the user’s knowledge, with the sole aim of quietly collecting credit card information using some NFC skimming technology [70].

**Cloning:** This involves replicating an NFC-enabled device to create a duplicate of all the card owner’s personal data [57,71]. The attacker can use cloning to take a series of premeditated criminal steps on the user’s card, reproducing the card to steal personal and financial data [29,72].

### 4.1. Cyber Risks Associated with NFC Transactions

The risk might expose an individual or organization to potential threats that can have serious adverse effects on such an individual or organization [73]. Although the number of NFC transactions is often limited by payment service providers (e.g., banks), there are still many cyber risks that exist [74]. Thus, risks must be assessed, and measures must be considered to mitigate NFC-related cyber risks. These measures become significant in financial processes, specifically when initiating and approving transactions [75]. Risk is an unavoidable term that affects business and financial activities [56].

In a bid to use more novel and effective means of payment, mobile payment was considered a more convenient and easy-to-use form of payment, though it is limited in acceptance among merchants and businesses because this mobile payment equipment might not be widely available, limiting payment options to businesses and users, respectively [76,77]. Notwithstanding the benefits of this payment option, it still has the potential for attacks, such as credit card fraud or skimming, stealing credit card information (such as card number, security code or CVV, and expiration date) from an unsuspecting user, for instance by remotely reading their NFC card/device information [60].

Despite the progress on novel systemic risk measures, research on the dynamics of systemic risk network structure and its community effect is still in its initial state, leaving many researchers with the need to investigate more reliable measures [78]. As a result, performing transactions with NFC features requires assessing all existing lacunae in the system that may bring risks to financial processes. Regardless, performing transactions over NFC platforms is still much poised with insurmountable and immeasurable attendant risks. These risks include cyber security, privacy, and fraud risks.

#### 4.1.1. Security Risks

The existing NFC systems have advanced security features, making them more secure than traditional EMV card transactions. However, many users are still left exposed to scams, fraud, and other criminal financial exploitation over the use of this technology [79,80]. Questions arise from many users, terminal operators, and other key interested parties about how exactly the NFC falls victim to security risks.

The device, though sleek and permeable in its design, functions effectively in the proximity of devices [36], allowing a free NFC up-to-date terminal that transmits information through two-way encryption and multi-factor authentication with biometrics or passcode, providing sufficient shielding of information from any potential fraudsters or attackers [80]. Nevertheless, the NFC payments have loopholes that allow security breaches due to their vulnerable hardware and software design. These allow transactions to be performed over metered networks and, seldom times, over unsecured and volatile wireless networks, which may expose the users unnecessarily to potential attacks [36].

Security risks often occur when a user unknowingly interacts with a compromised point-of-sale (POS) device or ATM. These potential risks raise concerns among users about the security architecture of NFC technology [17,81], as NFC-enabled devices can be vulnerable to cyber-attacks, theft, and data breaches, which can cause users of the technology to experience a lot of financial loss [82]. The following are possible classifications of security threats that may affect NFC transactions:

- Data breaches can happen in various forms, which can be grouped into data tampering, data interception/eavesdropping, and relay attacks [83,84]. Data tampering is a method used by criminally minded persons to manipulate data transmitted in an NFC transaction process with the aim of modifying data details to redirect funds from owners’ bank accounts to their own accounts [85]. Data tampering can occur using data corruption methods, which allows data communication processes to be flooded with corrupt information in a bid to breach the financial data transmitted over an NFC platform [66,86]. Data tampering can happen by data modification or insertion [87]. Data modification is a process that allows the attacker can alter and modify actual data into incorrect data during a data transmission process over an NFC network [17]. Additionally, the data insertion process involves a process where the attacker includes invalid data as part of the original data during a data transmission process over an NFC platform [88].
- Data interception and eavesdropping, a rather different yet similar security risk term, is explained as a method employed by criminally minded persons to tap into information, listen and see all financial communication transmitted between devices over NFC transaction platforms, to have unauthorized access to users’ financial information [89,90]. Other cyber threats have similar approaches and goals, such as relay attacks, which are similar to data interception threats [91,92]. The relay attack, which is a man-in-the-middle type attack, not only involves listening to data communication between devices but also involves the interception of data communication signals in an NFC transaction process to mislead others into believing that the main device owner initiated the transaction [92,93,94]. This type of data breach is carried out to gain unauthorized access to the user’s financial information to commit financial crimes [95].
- Unauthorized access allows persons with no right to financial information to gain unpermitted access to users’ financial data through the NFC transaction process. This can include any access to restricted devices or users’ mobile devices with the intent of initiating a transaction or committing financial crimes over NFC platforms [96,97]. However, the unauthorized access security issues are relevant to several other NFC cyber issues, as many other attacks start with unauthorized access.
- Identity theft is a process employed by cybercriminals to steal an individual’s payment-sensitive information, including, but not limited to, credit card information such as card expiration numbers, transaction codes, user access pins, and more [98]. Identity theft could occur due to the theft or loss of a user’s device into the hands of criminally minded persons, who take advantage of the device to make purchases or payments for transactions [99].

#### 4.1.2. Privacy Risk

Devices like the NFC allow users to interface with the technology by exchanging sensitive data like payment information, personal identification data, and location details [100,101]. Users want to ensure that their personal and financial information is always safe while using NFC technology. Privacy risk is a potentially severe issue that can affect the use of the NFC payment system. This risk emanates from the use of NFC-enabled tags to perform transactions. For the risk to occur, a tag can be placed on an individual or property without prior knowledge, exposing their information to a third party [19,102]. NFC transactions are susceptible to privacy threats, as financial information can be stolen over NFC transaction platforms (like NFC-enabled mobile wallets and POS terminals) [19,103]. The following are the classifications of privacy risks that can occur when using NFC technology.

**Data collection and tracking:** It is an essential feature of NFC technology, allowing users’ financial and personal data to be collected while using it [21,104,105]. The NFC architecture includes an asset tracking framework that provides storage benefits to businesses, helping them to use NFC tags in storing data in different formats, allowing the technology to be connected to a wide range of activities [106,107]. NFC uses radio waves of 13.56 MHz within a close range to transfer data but still allows attackers to use a sniffing coil to capture the signals transferring between the NFC reader and the tag [91]. Thus, NFC technology is left with data exposure risk, allowing attackers to collect and track users’ information while the NFC technology is in use.

**Personal information exposure:** Payment service providers and other involved organizations should only collect and store the users’ necessary personal information. Most of the time, users’ personal information only survives and is not exposed to third parties due to the NFC’s technological design and personal data protection regulations. NFC users might lose or find their personal and financial information exposed to third parties using certain platforms or devices or when their device is lost [108,109]. This exposed information can be utilized and processed wrongly to commit heinous crimes if it falls into the hands of criminals to commit financial fraud or other crimes [109,110,111].

#### 4.1.3. Fraud Risk

As mentioned earlier, NFC technology brings many benefits to its users, yet it leaves them vulnerable to possible fraud, legal liabilities, financial loss, and many other illegal, fraudulent transactions performed by criminals without clearance or authorization from the device owners [90]. Contactless fraud, for instance, can occur over the use of NFC technology in the form of an accidental loss or deliberate theft of a debit or credit card by criminals [112]. These stolen debit or credit cards can be used to make several limitless purchases or transactions before a PIN or a confirmation can be requested [113,114]. It is evident that fraud is a prevalent issue with contactless payments and can come in different forms, such as counterfeit transactions, chargeback fraud, and skimming and spoofing attacks.

**Counterfeit transactions** are illegally conducted transactions over NFC platforms. Counterfeit transactions are any transaction where criminals exploit the weaknesses in a system to outrun the interests of the users or device owners [90,115]. Counterfeit transactions can be performed using fake or cloned cards to store stolen card information and transmit it to perform payment or purchase transactions [83]. These transactions are rather increasing on the global map; despite the serious steps taken by the NFC technology designers to contain fraudulent occurrences over the device, many fraudsters have begun to adopt more complex strategies to gain access to the users’ financial and personal details. There are, however, several RFID and NFS anti-counterfeiting solutions that exist to tackle this issue [63,116].

Counterfeit transactions can occur through a series of activities like the shift in magnetic stripe liability, illegally prompted and approved transaction PIN, poor payment network rules, and lost or stolen device liability shift, which applies to only legitimate cards lost or stolen [117,118,119].

**Chargeback fraud** is another fraud risk that occurs with NFC technology; though it might be slightly considered a major risk in using NFC devices, it cannot be overlooked. Chargeback fraud occurs when a device user makes a purchase transaction with their own devices or cards and then requests a refund from the issuing bank after receiving the purchased items [120]. The idea painted by the card user for a refund request is that payment has been successfully made for a service or commodity, but the service or commodity has not been received when, indeed, the service or commodity has been received [119,121].

This is indeed a deceitful act and can be flagged as fraudulent by the cardholder. Chargeback fraud can be intentional or accidental. It can also be a legitimate or illegitimate claim for a refund by a customer for a transaction for which payment was made and was successful [57].

**Skimming and spoofing attacks**: The constant increase in technological advancement and payment mediums has necessitated increased cybercrimes, such as spoofing and skimming attacks [122,123]. Card skimming and phishing attacks have some similarities, e.g., in stealing users’ data. Phishing is a way of arbitrarily sending illegal emails that look legitimate to different email users to convince them to commit illegal transactions or to reveal sensitive and confidential information. Card skimming is described as a way of collecting users’ personal and financial data from payment cards without the user’s knowledge [124].

Skimming can either be physical, occurs through payment terminals or cards, or digital, known as a MageCart attack, which occurs through skimming of URLs, phishing emails, or remote infesting of e-commerce websites and apps to steal both user’s personal and financial data during a transaction [125,126,127]. To give a clearer view of the ideas of skimming, it is explained as a well-organized process used by skimmers to illegally collect users’ financial information, which can be used to perform unauthorized transactions. This organized process involves the installation of the skimming device as part of a normal card swipe mechanism in stores, gas stations, or points of purchase to steal information whenever there is a card swipe or use by device-by-device users. As explained further, this card swipe mechanism enables the skimmers to use this device to steal and store users’ information to perform transactions without the consent of the original card or device owners [112,128].

Skimming attacks look like a simple activity where criminals attach physical devices to card readers to capture the magnetic stripe data. However, these simple methods changed as technology advanced, leaving skimmers to develop new skimming skills that leverage stealing digital information remotely without close contact with the device itself and using other advanced techniques [122,129]. Spoofing, on the other hand, is explained as a criminal activity of gathering personal and financial information through impersonations or identity thefts [122]. It is described as a series of activities where a person or program poses as a legitimate entity or figure by falsifying data to gain an undue advantage through a process of convincing people to believe they are having protected communications with legitimate sources. To check for spoofing attacks, the correctness of the grammar will be checked alongside spellings, written sentences or phrases, the correctness of URLs, and misspellings of email sender addresses.

### 4.2. Payment Devices in NFC Transactions

Payment devices are devices used to initiate a physical or electronic purchase or payment for goods or services. Payment devices can take the form of a laptop, smartphone, payment card, or any other payment-enabled device, more particularly in NFC transactions [113]. The payment industry has been ardently working hard to eliminate continued usage of card transactions and encourage the use of contactless payment mediums like tap-and-pay credit and debit cards, which have been in existence since 2017. These contactless transactions are extremely modified to provide users with top-notch services while using NFC technology to make wireless transactions or at payment terminals [130,131].

Contactless cards such as the EMV, often known as “Chip & PIN”, have instantaneously become the lead card-based payment systems across the globe [130]. Smartphones, wearables, and contactless cards have been used in transactions, acting as the group of devices that can be reliably used in NFC transactions in the world market [30,132]. The value of the wearable device payment size was USD 55.8 billion in 2023 and is projected to experience an increase to USD 253.3 billion by 2032 [133]. This value increase shows the large adoption of NFC technology to meet the increasing demand for contactless payments for secure and safer payment transactions. As mentioned earlier in this paper, there are many NFC devices in the market. However, smart devices (e.g., smartphones) are currently the most common NFC readers [134].

### 4.3. Risk-Mitigating Strategies

Like any other risk, NFC-related risks must be assessed and mitigated for financial transactions over NFC platforms. Many research studies have suggested various ways to reduce payment risks over NFC platforms to the barest form [84,113,135]. In this study, we group risk-mitigating strategies to encryption and authentication techniques, fraud detection and prevention mechanisms, and regulatory compliance and standards.

#### 4.3.1. Encryption and Authentication Techniques

Two of the most common technical countermeasures in cyber security are securitizing communication channels and data using encryption and restricting access to systems and data using authentication techniques. By utilizing secure encryption techniques (e.g., currently AES 256 for encryption at rest), we can ensure that the data will not be accessed and/or modified in transit and where it is stored. Using authentication protocols and techniques, however, ensures that only authorized people can access the data and systems, and attack attempts like relay attacks and masquerading will not be successful [136,137]. The B2B and B2C transaction models encrypt customer data using elliptic curve cryptography (ECC) [36].

The authentication process involves using strict security measures, like MFA, to restrict access to technological devices used for payment over NFC platforms. Several techniques, such as message tokenization, aid in securing transactions initiated over the NFC platform [8,113]. The following are some methods that could be employed.

**Tokenization methods** are security measures used in NFC payments that send a token, one-time numbers that are randomly generated, associated with the user’s account, and can be used only once without giving access to valid credit card numbers. This validates a transaction (which can happen after authentication) instead of sending the actual credit card number for confirmation [72]. Secure transmission of these tokens between devices enables offline P2P payments, which is very helpful even when no internet is available [138]. Tokenization helps protect NFC-based transactions against man-in-the-middle, NFC relay, eavesdropping, and active relay attacks [139].

**Biometric authentication** is a security technique that allows users’ biometric characteristics to be utilized to authenticate them and gain access to a particular device [140]. It uses physical and complex security characteristics, such as fingerprints or facial recognition, to verify a user’s identity while using a device [141]. It is a high-value security framework that allows restriction to any other access due to the use of key body features that cannot be replaced or tampered with easily because everyone possesses unique physical body features (such as eyes and fingers) that differentiate us from one another. This type of security measure is not the only security layer to be provided, but it can be an additional security layer to prevent undue unauthorized access to attackers. Thus, solutions like multi-factor authentication (MFA), which is a combination of two or more authentication methods (e.g., PIN and biometrics), can make the authentication process more accurate [142].

When considering how to develop security measures to solve NFC payments security issues, there is no one-size-fits-all solution because each security measure might have a specific purpose and has its advantages and disadvantages. Some solutions combine various techniques to increase NFC-related transaction security; for instance, a study implements authentication protocols that operate via RFID and NFC communication between a server, an ATM, and a smartphone equipped with a secure element [140]. These combined techniques can be seen in smart devices such as the iPhone (i.e., Apple Pay), which employed the use of strict security measures to restrict undue access with biometric authorization and a one-time password to authenticate transactions over NFC platforms [143,144]. There are also other integrated security solutions for digital payments, such as using blockchain technology with NFC to increase the security of digital payments [22].

#### 4.3.2. Fraud Detection and Prevention Solutions

These are processes deployed to detect the occurrence of fraudulent activities and all other potential risks an individual may be exposed to over the NFC platforms and mechanisms to prevent its further occurrence [142,145,146]. In a revolving world where technologies are regularly updated, fraud can happen to anyone at any time while using any of the recent technologies, such as the NFC technology. To prevent or limit this occurrence, fraud detection and prevention solutions use various techniques such as user behavioral analysis, strong customer authentication, and real-time transaction monitoring, together with advanced fraud detection using ML and AI [97,114,147,148].

#### 4.3.3. Regulatory Compliance and Standards

There are NFC-related rules and regulations for merchants, payment service providers, and solution developers [97,149,150]. There are also upcoming standards for NFC; for instance, NFC Forum is developing a standard name, NFC DPP, to support the emerging European Union’s sustainable product regulations [151]. Many of these regulations and compliances require all the involved parties to ensure that NFC cards, devices, and transactions’ personal data will be kept and transferred secure (e.g., comply with GDPR), payment methods are accessible to the users with special needs (e.g., disabled customers), fraud detection/prevention solutions are in place, and agreements with payment service providers contain necessary security and privacy requirements [152].

There are also NFC-related standards that focus on specific aspects and areas of contactless payments. For instance, PCI (Payment Card Industry) Contactless Payments on COTS (CPoC™) specify security and data protection requirements that merchants can use to accept payments using NFC-enabled mobile devices [153].

**Figure 3:**
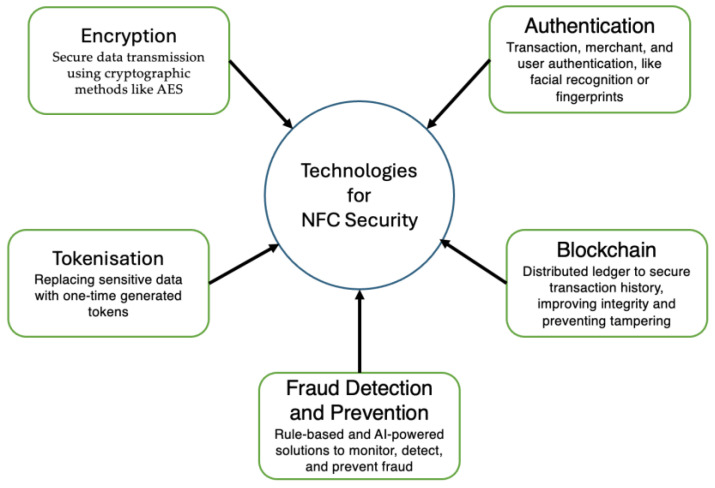
High-resolution block diagram of key NFC security technologies.

Many cybersecurity solutions can help tackle NFC-related cyber risks; however, Figure 3 shows some of the key technologies for NFC security.

### 4.4. Cybersecurity Solutions by World-Renowned Cybersecurity Companies

Several world-renowned cybersecurity companies provide robust solutions to mitigate the risks associated with NFC-enabled transactions. These solutions help protect users, merchants, payment service providers, and other businesses from threats such as data breaches, skimming, eavesdropping, unauthorized access, etc. The following are examples of solutions from world-renowned cybersecurity companies that secure NFC transactions.

Symantec (now NortonLifeLock) offers comprehensive mobile security solutions that protect NFC transactions by encrypting data transmission between devices and ensuring secure authentication protocols. Their Symantec Endpoint Protection Mobile (SEP Mobile) includes features like anti-malware, OS vulnerability prevention, real-time threat protection, and encryption to prevent data theft during transactions [154]. These solutions safeguard financial information transmitted via its security feature, which makes NFC contactless payments secure.

McAfee’s cybersecurity solutions provide advanced encryption and multi-factor authentication systems, which are essential for securing NFC-enabled devices such as smartphones and wearables [155]. For instance, McAfee’s ATR identified a design vulnerability that could enable an attacker to clone McLear NFC Ring and access a home with an NFC-enabled smart lock [156]. The NFC rings can also be used for payments, so the ART solution can be considered a useful NFC security solution in general [157]. By encrypting data at rest and in transit, McAfee protects against interception attacks during NFC-based transactions. McAfee’s machine learning algorithms also detect anomalies and fraudulent activities, ensuring secure payments.

Kaspersky’s mobile security solution focuses on fraud detection and real-time monitoring of transactions [158]. Their products, such as Kaspersky Embedded Systems Security, can detect malicious activity, ensuring that NFC transactions are conducted securely [159]. Kaspersky also offers tokenization solutions, ensuring that sensitive data are replaced with unique tokens during NFC payments, minimizing the risk of unauthorized data access [160].

Cisco’s network security solutions offer end-to-end encryption and secure communication channels, ensuring that NFC-enabled devices are protected from external threats. Cisco’s security architecture integrates blockchain technology to provide added layers of protection for transaction histories, ensuring data integrity and preventing tampering in NFC-enabled payment systems [161].

IBM’s blockchain solution provides blockchain-based security frameworks for payment systems [162]. IBM Safer Payments is an AI-powered solution that helps create decision models for adapting to emerging cyber threats and detecting frauds more quickly and accurately [163].

Foresiet Xtreme, a proactive threat intelligence solution, provides a digital risk protection SaaS platform. This solution can help us detect NFC-targeted attacks, such as malware, which can steal NFC data from mobile phones [164].

## 5. Discussion

Discovering ways of mitigating payment issues occurring over the NFC payment platforms was the main objective of this study research. The study adopted an in-depth SLR collected from 2017 to date and delved into ways to understand better how to eliminate this long-standing issue within the NFC payment platforms. During the pursuit of this objective, the study was able to establish that the NFC really possesses features that have well made a pathway and revolution in the way businesses will be done globally before this time. Further development in this regard will provide more modalities and developments in business. The customers and merchants using NFC technology must be confident that the payment platform is safe.

To establish a renewed and unrivaled level of confidence in payment forms and mediums explored by customers, merchants, and even solution designers, this study described the main ideologies that may be stumbled upon while trying to understand the NFC payment environment. These main ideologies included RFID, NFC-enabled devices, and many other technological concepts. The study deepened the knowledge of its host readers into an understanding of each NFC payment device and the risks that users of these devices are susceptible to falling victim to while transacting in an NFC payment environment.

Nevertheless, identifying problems alone was not the only focus of the study, as the study adopted the system of identifying the problems and then the existing solutions to solve them. Accordingly, the study presented possible solutions to see to the end of these many issues experienced while performing transactions over the NFC platform. Based on the compiled works of other authors, each of these suggested solutions has proven more effective than usual, leaving more records of accuracy in other experienced cases. The study, therefore, opines that adopting these ideologies into the NFC payment platform will do better by making the system overly reliable for users and merchants to bank. As noted, the technology is still at its refinement stage, and therefore, there is room for further research and modifications in the near future to avoid total replacement of the technology in the face of highly intensive competition.

### 5.1. Challenges and Opportunities in NFC Transactions Security

Performing transactions over the NFC transaction is challenged by frequent privacy and security risks that affect users’ personal and financial information while transacting over NFC platforms. The NFC possesses opportunities to stay in business if it takes full advantage of developing a serious security architecture that protects its users from falling victim to these possible threats. The NFC will be better improved and provide sufficient quality assurance to its users if it adopts, as part of its features, the use of blockchain and distributed ledger technology to improve its security architecture.

We can conclude that NFC payment devices are very effective means of payment. Though confronted with several threats, if adequately mitigated, the payment systems will be safe and reliable for users to bank upon while making payments for transactions.

This study’s implications for research and practice are that it provides researchers, device makers, users, and other stakeholders with technical knowledge about mitigating risks in NFC payment transactions. It is also a contribution to knowledge and a basis for other research works to be conducted in the future. The implication, in the long run, is that previous research held on the subject matter will be rendered inapplicable, as this study helps to highlight several flaws in previous literature, therefore providing a basis for more research that aligns with the latest trends and developments arising on the NFC payment technology Furthermore, the study research awakens researchers to smear up this latest development with the technology while providing better means of seamless yet save platforms for transactions that users can trust.

This study provides a basis of knowledge to NFC technology’s stakeholders through a careful highlighting and analysis of the existing flaws in the NFC payment system that can lead to the loss of users’ hard-earned incomes. The study also gives a cursory view rooted in the information collected from a careful SLR about how risks in NFC payment technology can be mitigated.

### 5.2. Recommendations to Stakeholders

Based on this study’s findings, the following recommendations are for NFC stakeholders, such as payment service providers, NFC device vendors, and cybersecurity experts. Considering these recommendations can further enhance the security of NFC-enabled transactions and platforms.

**Adoption of Multi-Layered Security Protocols**: Stakeholders, including financial institutions, merchants, and service providers, should implement multi-layered security protocols for NFC-enabled systems. Combining multiple security layers, such as encryption, tokenization, and biometric authentication, can significantly reduce the risk of attacks such as card skimming, cloning, and relay attacks. Implementing multi-layered security protocols can prevent several cyber-attacks that exploit NFC vulnerabilities, such as CWE-287, which could cause “unauthorized tampering of device configuration over NFC communication” (CVE-2024-0568) [165].

**Biometric Authentication Integration**: NFC-enabled devices should increasingly incorporate biometric authentication (if it is practical), including fingerprint scanning and facial recognition, to add an extra layer of protection. Biometric features are more difficult to replicate than traditional PIN-based systems and can prevent unauthorized access to payment platforms. For instance, an NFC vulnerability (CVE-2019-9295) allows unauthorized NFC access due to insufficient user authentication when an NFC tag initiates a connection [166]. This vulnerability allowed attackers to exploit the NFC stack to execute actions on the device without explicit user consent if NFC was enabled. The biometric authentication integration prompts users to authenticate before allowing access to certain NFC-based features, mitigating the risk of unauthorized actions through NFC.

**Real-Time Monitoring and Fraud Detection**: Security experts should ensure that real-time transaction monitoring systems are deployed to detect and prevent fraudulent activities as they happen. Integrating AI-powered anomaly detection and machine learning algorithms can help identify suspicious patterns and block unauthorized transactions before they are completed. For instance, an NFC vulnerability (CVE-2020-0022) affects Android devices, allowing remote code execution (RCE) via NFC without user interaction [167]. This vulnerability in Android’s Bluetooth and NFC stack allowed attackers to execute code remotely through maliciously crafted NFC messages. Implementing real-time monitoring and fraud detection can help mitigate this vulnerability by detecting unusual NFC activities and quickly flagging or blocking potentially malicious transactions. Such solutions can alert users or administrators when suspicious NFC interactions are detected, helping prevent unauthorized access and potential exploitation of vulnerabilities like this one.

**Use of Blockchain for Secure Transaction History**: Stakeholders should consider leveraging blockchain technology to secure transaction histories in NFC payments. Blockchain’s decentralized nature protects against tampering and fraud by ensuring that transaction records remain immutable and transparent to all parties involved. For instance, an NFC vulnerability (CVE-2023-46765) involves uncaught exceptions in the NFC module, which, if exploited, can affect the availability of NFC [168]. Integrating blockchain technology to maintain a secure and immutable transaction history can help detect and prevent unauthorized or anomalous NFC transactions. Recording each NFC transaction on a blockchain can promptly identify any irregularities, and the transparent ledger ensures that all transactions are traceable and verifiable. Thus, this solution can enhance security and mitigate the impact of such vulnerabilities.

**Deployment of End-to-End Encryption**: End-to-end encryption should be mandatory for all NFC-enabled payment systems, ensuring that data are protected both in transit and at rest. Encryption protocols such as AES-256 and elliptic curve cryptography (ECC) should safeguard sensitive information from eavesdropping and unauthorized access. For instance, an NFC vulnerability (CVE-2019-13143) affects NFC communication by exposing it to potential eavesdropping and interception attacks [169]. It can allow attackers to intercept and potentially modify data transmitted over NFC. Encrypting data from the sender to the receiver ensures that only authorized devices can decrypt and read the information. This approach would prevent attackers from accessing or tampering with data, even if they manage to intercept the communication.

**Tokenization for Data Protection**: Implementing tokenization in NFC systems is crucial for protecting cardholder data. Businesses can significantly reduce the likelihood of data breaches by replacing sensitive information, such as credit card numbers, with tokens. These tokens should only be usable for specific transactions, making it impossible for attackers to reuse stolen data. For instance, an NFC vulnerability (CVE-2023-35671) allows unauthorized NFC devices to access credit card details under specific conditions [170]. This vulnerability allowed attackers to exploit a loophole in Android’s Screen Pinning feature, enabling NFC devices to skim credit card details from Google Wallet without proper authorization. Implementing tokenization can mitigate this vulnerability by replacing sensitive credit card information with unique tokens during NFC transactions [171]. These tokens are meaningless if intercepted, as they cannot be reverse-engineered to reveal the original credit card details. Using tokenization, even if an attacker intercepts the token, it cannot be used for fraudulent transactions, enhancing data security during NFC communications.

**Enhanced Public-Key Infrastructure (PKI)**: Stakeholders should adopt enhanced PKI systems to ensure secure data exchange between NFC-enabled devices and servers. More robust PKI implementations with certificate-based authentication can help prevent impersonation attacks and ensure that only trusted devices and servers participate in the transaction. A recent NFC vulnerability (CVE-2024-38381) affects the Linux kernel’s NFC subsystem, specifically within the nci_rx_work() function, due to insufficient validation of packet headers [172]. This flaw could allow an attacker to send malicious packets, resulting in unauthorized access or system instability. PKI provides a framework for managing digital certificates and public-key encryption [173], ensuring that only trusted devices can communicate over NFC. By validating the authenticity of devices and encrypting data transmissions, PKI helps prevent unauthorized access and data tampering, which can address vulnerabilities like CVE-2024-38381.

**Regular Security Audits and Compliance Checks**: Organizations using NFC payment platforms should perform regular security audits and compliance checks to identify vulnerabilities and ensure that their systems meet the latest regulatory standards. Adherence to guidelines such as PCI-DSS (Payment Card Industry Data Security Standard) [153] can help mitigate risks. For instance, the CVE-2023-46765 vulnerability involves uncaught exceptions in the NFC module that can affect NFC availability [168]. Conducting routine security assessments can detect such issues early, ensuring timely remediation and maintaining the integrity of NFC functionalities.

**Consumer Education and Awareness**: Besides technical measures, stakeholders should invest in educating consumers on safe NFC usage. This includes informing users about the risks of tapping their devices on unverified readers and encouraging the use of additional security features, such as two-factor authentication (2FA), when available. Mobile device users should know that installing any unknown app on their mobile phones can put them at risk of losing money, as the apps can make their phones vulnerable. For instance, an NFC beaming vulnerability can bypass security controls in Android (CVE-2019-2114) [174].

**Collaboration with Cybersecurity Firms**: Companies providing NFC-based payment solutions should establish collaborative partnerships with world-renowned cybersecurity firms to stay ahead of emerging threats. By leveraging the expertise of leading security companies, organizations can access the latest threat intelligence and security updates to protect their systems. A recent NFC vulnerability (CVE-2024-24313) involves an Insecure Direct Object Reference (IDOR) flaw, which allows unauthorized access to sensitive information through NFC-enabled applications [175]. This vulnerability permits attackers to access unauthorized resources by manipulating input parameters in NFC-enabled applications, potentially leading to data breaches and unauthorized transactions [176]. Collaborating with cybersecurity firms enables organizations to conduct thorough security assessments, identify vulnerabilities like CVE-2024-24313, and implement appropriate mitigations. These firms provide expertise in penetration testing, code review, and security best practices, ensuring that NFC applications are robust against potential threats.

### 5.3. Study Limitations

The first limitation is that NFC technology is still emerging, and it represents a new field of research for academics and experts focused on its security and privacy challenges. Despite this, we believe the current literature review is comprehensive. The second potential limitation can be the possibility of subjectivity in selecting the research papers for inclusion. However, efforts were made to review and assess the chosen papers to ensure more objective decisions.

### 5.4. Contributions to Knowledge

The study provides an improvement in knowledge and understanding of cybersecurity issues that relate to mobile payments, particularly security and privacy risks in NFC transactions. The study through an in-depth SLR approach aided us, researchers, tech experts, governments, and business partners in better understanding the payment options available in NFC transactions, their individual advantages and disadvantages, their vulnerabilities that limit their effectiveness, and ways to mitigate against the further occurrence of these risk factors in payment procedures, in a bid to efficiently protect user’s personal and financial information. The study assisted us further in properly gaining insights into users’ perceptions and behaviors regarding NFC payments. Understanding users’ attitudes and adoption patterns better inform the design of more user-friendly and secure payment systems, enhancing the overall user experience and promoting greater trust in digital financial technologies.

### 5.5. Future Studies

In previous studies, the NFC security framework has provided a tight security architecture. However, it is enveloped by slight security vulnerability due to the rise in technological developments and the increase in malefactor knowledge regarding violating NFC transactions through available loopholes. In subsequent research, NFC should bring forth technological development that presents a tough multi-layered security architecture (e.g., based on a Defense-in-Depth approach) to prevent third-party access to users’ personal and financial information. It should include its ability to provide a more reliable and advanced form of technology that matches the constant updates in technological advancement. Machine learning and artificial intelligence (AI) could be adopted into the designs of devices with NFC security-related features and cyber threat protection of e-payment service providers to address the NFC’s existing payment risks.

## 6. Conclusions

This study confidently concludes that NFC payment technology represents the new age; despite its many identified flaws, it is considered one of the best payment technologies offered to mankind. Adopting all the identified solutions to its architectural and systemic makeup will contribute sufficiently to its effective capacity and the level of trust that users of this technology have in it.

This paper has undertaken a thorough investigation into the cyber risks associated with NFC transactions and the strategies available for mitigating these risks, particularly focusing on payment devices. Through the systematic literature review, we have gained insights into NFC technology, security vulnerabilities, privacy concerns, and fraudulent activities. The analysis of mitigation strategies such as encryption, authentication, tokenization, biometric authentication, and fraud detection methods has provided valuable understanding for stakeholders in the field of NFC transactions. The findings underscore the significance of addressing these security risks and offer recommendations for stakeholders in the domain. Overall, this study contributes to the ongoing discourse on NFC transaction security and provides a foundation for future research and industry actions to bolster the security of NFC payment solutions.

## Figures and Tables

**Table 1:** Previous survey studies.

Sources	Focus	Major Contribution	Shortcomings
[20]	Mobile payment security	Examining the NFC vulnerabilities and suggesting some mitigation techniques	Lacks a comprehensive exploration of advanced methods like biometric authentication or detailed practical applications of multi-layered defense strategies. It does not provide in-depth analysis of NFC-specific cyber risks/attacks, such as skimming, cloning, and malware attacks
[19]	NFC device security and privacy	Identified early risks like eavesdropping and interception	No in-depth analysis of advanced fraud detection methods. Limited focus on evolving attacks such as relay attacks
[21]	NFC technology and payment processes	Reviewed technological frameworks for NFC payments	Lacks a deep dive into specific mitigation strategies beyond authentication and encryption
[8]	NFC security policies and countermeasures	Detailed exploration of contactless smart card systems and attacks	Did not explore more advanced techniques like biometrics and the related vulnerabilities
[16]	Trust and security perception in NFC mobile payments	Analyzed trust and perceived risks influencing NFC payment adoption	Lacked technical exploration of specific security measures against attacks. It also lacks coverage of advanced security mitigations such as AI and blockchain solutions
[22]	Blockchain in NFC payments	Explored blockchain’s role in improving NFC payment security and efficiency	Did not deeply analyze user-centered threats like phishing or skimming

## References

[1] Min H., Galle W.P. (1999). Electronic commerce usage in business-to-business purchasing. Int. J. Oper. Prod. Manag..

[2] Grewal R., Lilien G.L., Bharadwaj S., Jindal P., Kayande U., Lusch R.F., Mantrala M., Palmatier R.W., Rindfleisch A., Scheer L.K. (2015). Business-to-business buying: Challenges and opportunities. Cust. Needs Solut..

[3] Malherbe M., Simon F. (2021). Near-field communication (NFC) technology emergence: One or several technological paths?. J. Innov. Econ. Manag..

[4] Lacmanović I., Radulović B., Lacmanović D. Contactless payment systems based on RFID technology. Proceedings of the 33rd International Convention MIPRO.

[5] Kulkarni R. (2021). Near field communication (NFC) technology and its application. Techno-Societal 2020: Proceedings of the 3rd International Conference on Advanced Technologies for Societal Applications—Volume 1.

[6] Chandrasekar P., Dutta A. (2021). Recent developments in near field communication: A study. Wirel. Pers. Commun..

[7] Pasquet M., Reynaud J., Rosenberger C. “Payment with mobile nfc phones” how to analyze the security problems. Proceedings of the 2008 International Symposium on Collaborative Technologies and Systems.

[8] Gupta B.B., Narayan S. (2020). A survey on contactless smart cards and payment system: Technologies, policies, attacks and countermeasures. J. Glob. Inf. Manag. (JGIM).

[9] Vidakis K., Mavrogiorgou A., Kiourtis A., Kyriazis D. A comparative study of short-range wireless communication technologies for health information exchange. Proceedings of the 2020 International Conference on Electrical, Communication, and Computer Engineering (ICECCE).

[10] Ali A.H., Abouhogail R.A., Tarrad I.F., Youssef M.I. (2014). Assessment and comparison of commonly used wireless technologies from mobile payment systems perspective. Int. J. Softw. Eng. Its Appl..

[11] Hamzah M.L., Desnelita Y., Purwati A.A., Rusilawati E., Kasman R., Rizal F. (2019). A review of Near Field Communication technology in several areas. Rev. Espac..

[12] Pulipati M., Phani S. (2013). Comparison of various short range wireless communication technologies with nfc. Inter. J. Sci. Res.

[13] Luo J.-N., Yang M.-H. (2019). EMV-compatible offline mobile payment protocol with mutual authentication. Sensors.

[14] Kairo J. (2024). Future of paying? Exploring the possibilities of contactless payment with Ultra-Wideband technology. Comput. Sci..

[15] Orman I., Teker D., Teker S. (2022). Evolution of digital payment systems and a breakthrough. J. Econ. Manag. Trade.

[16] Alrawad M., Lutfi A., Almaiah M.A., Elshaer I.A. (2023). Examining the influence of trust and perceived risk on customers intention to use NFC mobile payment system. J. Open Innov. Technol. Mark. Complex..

[17] Almaiah M.A., Al-Rahmi A., Alturise F., Hassan L., Lutfi A., Alrawad M., Alkhalaf S., Al-Rahmi W.M., Al-sharaieh S., Aldhyani T.H. (2022). Investigating the effect of perceived security, perceived trust, and information quality on mobile payment usage through near-field communication (NFC) in Saudi Arabia. Electronics.

[18] Clarisa D., Marlena D. Design of secure NFC e-payment with ambient conditions-based solutions and Chaskey algorithm. Proceedings of the 2021 6th International Workshop on Big Data and Information Security (IWBIS).

[19] Madlmayr G., Langer J., Kantner C., Scharinger J. NFC Devices: Security and Privacy. Proceedings of the 2008 Third International Conference on Availability, Reliability and Security.

[20] Bojjagani S., Sastry V.N., Chen C.-M., Kumari S., Khan M.K. (2023). Systematic survey of mobile payments, protocols, and security infrastructure. J. Ambient Intell. Humaniz. Comput..

[21] Shobha N.S.S., Aruna K.S.P., Bhagyashree M.D.P., Sarita K.S.J. NFC and NFC payments: A review. Proceedings of the 2016 International Conference on ICT in Business Industry & Government (ICTBIG).

[22] Mandal S., Shill P.C. Ensuring Security and Efficiency in Digital Payments using Blockchain with NFC. Proceedings of the 2024 International Conference on Knowledge Engineering and Communication Systems (ICKECS).

[23] Saunders M. (2009). Research Methods for Business Students.

[24] Moher D., Liberati A., Tetzlaff J., Altman D.G. (2010). Preferred reporting items for systematic reviews and meta-analyses: The PRISMA statement. Int. J. Surg..

[25] BasuMallick C. (2022). What Is NFC (Near Field Communication)? Definition, Working, and Examples. https://www.spiceworks.com/tech/networking/articles/what-is-near-field-communication/.

[26] Linn H., Nutting R. (2022). CompTIA PenTest+ Certification All-In-One Exam Guide, (Exam PT0-002).

[27] Want R. (2011). Near field communication. IEEE Pervasive Comput..

[28] Beal V. (2024). Near Field Communication (NFC). https://www.techopedia.com/definition/27583/near-field-communication-nfc.

[29] Chen X., Choi K., Chae K. (2017). A secure and efficient key authentication using bilinear pairing for NFC mobile payment service. Wirel. Pers. Commun..

[30] Lathiya P., Wang J. (2021). Near-field communications (NFC) for wireless power transfer (WPT): An overview. Wireless Power Transfer—Recent Development, Applications and New Perspectives.

[31] Goitre A. (2020). Bringing NFC Wireless Charging to Consumer Devices.

[32] Kavitha T. (2022). Internet of Everything: Smart Sensing Technologies.

[33] Singh N.K. (2020). Near-Field Communication (NFC): An Alternative to RFID in Libraries.

[34] Cho J.H., Kim J., Kim J.W., Lee K., Ahn K.D., Kim S. An NFC transceiver with RF-powered RFID transponder mode. Proceedings of the 2007 IEEE Asian Solid-State Circuits Conference.

[35] Albattah A., Alghofaili Y., Elkhediri S. NFC Technology: Assessment Effective of Security towards Protecting NFC Devices & Services. Proceedings of the 2020 International Conference on Computing and Information Technology (ICCIT-1441).

[36] Bojjagani S., Sastry V.N. (2019). A secure end-to-end proximity NFC-based mobile payment protocol. Comput. Stand. Interfaces.

[37] Motola C. NFC Payment Guide: What Are NFC Mobile Payments & NFC Readers. https://www.merchantmaverick.com/what-is-nfc/.

[38] Giese D., Liu K., Sun M., Syed T., Zhang L. (2019). Security analysis of near-field communication (NFC) payments. arXiv.

[39] Ghosh S., Majumder A., Goswami J., Kumar A., Mohanty S.P., Bhattacharyya B.K. (2016). Swing-pay: One card meets all user payment and identity needs: A digital card module using NFC and biometric authentication for peer-to-peer payment. IEEE Consum. Electron. Mag..

[40] Marriott H.R., Williams M.D. (2018). Exploring consumers perceived risk and trust for mobile shopping: A theoretical framework and empirical study. J. Retail. Consum. Serv..

[41] Raina V.K. (2017). NFC Payment Systems and the New Era of Transaction Processing.

[42] Treece D. (2024). What Are NFC Mobile Payments? This Contactless Payment Option Can Boost Customer Convenience and Facilitate Seamless Transactions. https://www.businessnewsdaily.com/16250-nfc-mobile-payments.html.

[43] Dai D., An Z., Pan Q., Yang L. (2023). MagCode: NFC-Enabled Barcodes for NFC-Disabled Smartphones. Proceedings of the 29th Annual International Conference on Mobile Computing and Networking.

[44] Shin S., Lee W.-J. (2021). Factors affecting user acceptance for NFC mobile wallets in the US and Korea. Innov. Manag. Rev..

[45] Al-Amri R., Maarop N., Jamaludin R., Samy G., Magalingam P., Hassan N.H., Ten D.W.H., Daud S.M. (2018). Correlation analysis between factors influencing the usage intention of NFC mobile wallet payment. J. Fundam. Appl. Sci..

[46] McGrath S.K., Whitty S.J. (2017). Stakeholder defined. Int. J. Manag. Proj. Bus..

[47] Michael C. (2023). Stakeholders Outline Impact of Contactless Payment Across Sectors. https://businessday.ng/technology/article/stakeholders-outline-impact-of-contactless-payment-across-sectors/.

[48] Benyó B. Business process analysis of NFC-based Services. Proceedings of the 2009 IEEE International Conference on Computational Cybernetics (ICCC).

[49] Liébana-Cabanillas F., Molinillo S., Ruiz-Montañez M. (2019). To use or not to use, that is the question: Analysis of the determining factors for using NFC mobile payment systems in public transportation. Technol. Forecast. Soc. Chang..

[50] Li H., Liu Y., Heikkilä J. Understanding the factors driving NFC-enabled mobile payment adoption: An empirical investigation. Proceedings of the PACIS 2014 Proceedings.

[51] Management P.S., CBN (2023). Guidelines to Banks for Contactless Payments in Nigeria.

[52] Smets J., Ergeerts G., Beyers R., Schrooyen F., Ceulemans M., Wante L., Renckens K. An NFC-based customer loyalty system. Proceedings of the First International Conference on Mobile Services, Resources, and User.

[53] Gerpott T.J., Meinert P. (2017). Who signs up for NFC mobile payment services? Mobile network operator subscribers in Germany. Electron. Commer. Res. Appl..

[54] Reimers J., Honekamp W. (2020). Risk Analysis of NFC Payment Systems. Mobility in a Globalised World 2019.

[55] Ali A.H., Abouhogail R.A., Tarrad I.F., Youssef M.I. (2017). A new design of Mobile Payment system based on NFC Technology. Int. J. Eng. Technol. IJET-IJENS.

[56] Cremer F., Sheehan B., Fortmann M., Kia A.N., Mullins M., Murphy F., Materne S. (2022). Cyber risk and cybersecurity: A systematic review of data availability. Geneva Pap. Risk Insur.-Issues Pract..

[57] Vishwakarma P.P., Tripathy A.K., Vemuru S. (2019). An empiric path towards fraud detection and protection for NFC-enabled mobile payment system. Telkomnika.

[58] Markets R.a. (2024). Near Field Communication Enabled Handsets Global Strategic Business Report 2024–2030. https://finance.yahoo.com/news/near-field-communication-enabled-handsets-095100067.html.

[59] Sasu D.D. (2023). Payment Methods in Nigeria-Statistics & Facts. https://www.statista.com/topics/7133/payment-methods-in-nigeria/#topicOverview.

[60] Ye H., Lee C.-J., Wu T.-Y., Yang X.-D., Chen B.-Y., Liang R.-H. Body-Centric NFC: Body-Centric Interaction with NFC Devices Through Near-Field Enabled Clothing. Proceedings of the 2022 ACM Designing Interactive Systems Conference.

[61] Juen Y.W., Balachandran D. (2021). Predicting the diffusion of NFC-enabled smartphone payment in Malaysia. Int. J. Model. Oper. Manag..

[62] Ramos de Luna I., Montoro-Ríos F., Liébana-Cabanillas F.J., Management Association I.R. (2018). New Perspectives on Payment Systems: Near Field Communication (NFC) Payments Through Mobile Phones. Mobile Commerce: Concepts, Methodologies, Tools, and Applications.

[63] Anggoro O., Dzulfikar M., Purwandari B., Mishbah M. Secure Smartphone-Based NFC Payment to Prevent Man-in-the-Middle Attack. Proceedings of the 2019 International Conference on Informatics, Multimedia, Cyber and Information System (ICIMCIS).

[64] Li P., Fang H., Liu X., Yang B. A countermeasure against relay attack in NFC payment. Proceedings of the Second International Conference on Internet of things, Data and Cloud Computing.

[65] Yuvarani R., Mahaveerakannan R. Payment Security Expert: Analyzing Smart Cards and Contactless Payments with Cryptographic Techniques. Proceedings of the 2024 2nd International Conference on Sustainable Computing and Smart Systems (ICSCSS).

[66] Shariati S.M., Abouzarjomehri A., Ahmadzadegan M.H. Investigating NFC technology from the perspective of security, analysis of attacks and existing risk. Proceedings of the 2015 2nd International Conference on Knowledge-Based Engineering and Innovation (KBEI).

[67] Lakshmanan D., Nagoor Meeran A.R. (2017). NFC Logging Mechanism—Forensic Analysis of NFC Artefacts on Android Devices. Artificial Intelligence and Evolutionary Computations in Engineering Systems.

[68] Arabo A., Pranggono B. Mobile Malware and Smart Device Security: Trends, Challenges and Solutions. Proceedings of the 2013 19th International Conference on Control Systems and Computer Science.

[69] Kortvedt H., Mjolsnes S. Eavesdropping near field communication. Proceedings of the Norwegian Information Security Conference (NISK).

[70] Guers K., Chowdhury M.M., Rifat N. Card Skimming: A Cybercrime by Hackers. Proceedings of the 2022 IEEE International Conference on Electro Information Technology (eIT).

[71] Roland M., Langer J. Cloning Credit Cards: A Combined Pre-play and Downgrade Attack on {EMV} Contactless. Proceedings of the 7th USENIX Workshop on Offensive Technologies (WOOT 13).

[72] Chabbi S., Madhoun N.E., Khamer L. Security of NFC Banking Transactions: Overview on Attacks and Solutions. Proceedings of the 2022 6th Cyber Security in Networking Conference (CSNet).

[73] Zhao H., Anong S.T., Zhang L. (2019). Understanding the impact of financial incentives on NFC mobile payment adoption. Int. J. Bank Mark..

[74] Korhonen N.-P. (2017). NFC Payment & Security Threats. https://www.theseus.fi/handle/10024/125290?show=full.

[75] Dudin M.N., Zasko V.N., Frolova E.E., Pavlova N.G., Rusakova E.P. (2018). Mitigation of cyber risks in the field of electronic payments: Organizational and legal measures. J. Adv. Res. Law Econ..

[76] Świecka B., Terefenko P., Wiśniewski T., Xiao J. (2021). Consumer financial knowledge and cashless payment behavior for sustainable development in poland. Sustainability.

[77] Verkijika S.F., Neneh B.N. (2021). Standing up for or against: A text-mining study on the recommendation of mobile payment apps. J. Retail. Consum. Serv..

[78] Asanprakit S., Kraiwanit T. (2023). Causal factors influencing the use of social commerce platforms. J. Open Innov. Technol. Mark. Complex..

[79] Anusha R. (2018). Qualitative assessment on effectiveness of security approaches towards safeguarding NFC devices & services. Int. J. Electr. Comput. Eng. (IJECE).

[80] Al-Haj A., Al-Tameemi M.A. Providing security for NFC-based payment systems using a management authentication server. Proceedings of the 2018 4th International Conference on Information Management (ICIM).

[81] Kajol K., Singh R., Paul J. (2022). Adoption of digital financial transactions: A review of literature and future research agenda. Technol. Forecast. Soc. Chang..

[82] Liébana-Cabanillas F., Sánchez-Fernández J., Muñoz-Leiva F. (2014). Antecedents of the adoption of the new mobile payment systems: The moderating effect of age. Comput. Hum. Behav..

[83] Molitor D., Raghupathi W., Saharia A., Raghupathi V. (2023). Exploring Key Issues in Cybersecurity Data Breaches: Analyzing Data Breach Litigation with ML-Based Text Analytics. Information.

[84] Singh M.M., Adzman K., Hassan R. (2018). Near Field Communication (NFC) technology security vulnerabilities and countermeasures. Int. J. Eng. Technol..

[85] No P. Users’ Perception of NFC Technology in Digital Payment Transactions in Indonesia. https://ijefm.co.in/v6i5/15.php.

[86] Trautman L.J., Ormerod P.C. (2016). Corporate directors’ and officers’ cybersecurity standard of care: The Yahoo data breach. Am. UL Rev..

[87] Banerjee D. (2023). Data Tampering: A Comprehensive Guide. https://www.kosli.com/blog/data-tampering-a-comprehensive-guide/#:~:text=Data%20tampering%20is%20the%20deliberate,or%20any%20digital%20storage%20device.

[88] Haselsteiner E., Breitfuß K. Security in near field communication (NFC). Proceedings of the Workshop on RFID Security.

[89] Chattha N.A. NFC—Vulnerabilities and defense. Proceedings of the 2014 Conference on Information Assurance and Cyber Security (CIACS).

[90] Ayereby M.P.-M. (2018). Overcoming Data Breaches and Human Factors in Minimizing Threats to Cyber-Security Ecosystems.

[91] Wang Y., Zou J., Zhang K. (2023). Deep-Learning-Aided RF Fingerprinting for NFC Relay Attack Detection. Electronics.

[92] Imran M.I.I., Putrada A.G., Abdurohman M. Detection of Near Field Communication (NFC) Relay Attack Anomalies in Electronic Payment Cases using Markov Chain. Proceedings of the 2019 Fourth International Conference on Informatics and Computing (ICIC).

[93] TapTrack (2016). NFC Relay Attacks. https://taptrack.com/nfc-relay-attacks/.

[94] Akter S., Chellappan S., Chakraborty T., Khan T.A., Rahman A., Al Islam A.A. (2020). Man-in-the-middle attack on contactless payment over NFC communications: Design, implementation, experiments and detection. IEEE Trans. Dependable Secur. Comput..

[95] Rahmad N.N., Zullzaidi N.S.M., Azmi N.D.F., Khairudin N.M. Mobile Payment Security: A Critical Analysis of Vulnerabilities & Emerging Threats. Authorea Preprints, 2024. https://www.researchgate.net/publication/377716256_Mobile_Payment_Security_A_Critical_Analysis_of_Vulnerabilities_Emerging_Threats.

[96] Bojjagani S., Rao P.V.V., Vemula D.R., Reddy B.R., Lakshmi T.J. (2022). A secure IoT-based micro-payment protocol for wearable devices. Peer-to-Peer Netw. Appl..

[97] Faccia A. (2023). National payment switches and the power of cognitive computing against fintech fraud. Big Data Cogn. Comput..

[98] Opderbeck D.W. (2022). Cybersecurity and Data Breach Harms: Theory and Reality. Md. L. Rev..

[99] Saeed D., Iqbal R., Sherazi H.H.R., Khan U.G. (2019). Evaluating Near-Field Communication tag security for identity theft prevention. Internet Technol. Lett..

[100] Sahi A.M., Khalid H., Abbas A.F., Zedan K., Khatib S.F.A., Al Amosh H. (2022). The Research Trend of Security and Privacy in Digital Payment. Informatics.

[101] Kang J., Park J.H., Suk S. (2015). Design of a Distributed Personal Information Access Control Scheme for Secure Integrated Payment in NFC. Symmetry.

[102] Tang B., Baoguo Y., Fushun N., Peijun H. Review of Near-Field Microwave Microscopy Technology. Proceedings of the 2021 IEEE 6th International Conference on Computer and Communication Systems (ICCCS).

[103] Rajan M.A. (2011). The future of wallets: A look at the privacy implications of mobile payments. CommLaw Conspec..

[104] Hutagalung G.A., Dalimunte Y.A., Khairina I., Lubis M.a.Z., Firmansyah D., Sinaga D.N., Simanjuntak I.S., Indandi Z. (2024). Attendance Data Collection Using NFC Tags. Int. J. Res. Vocat. Stud. (IJRVOCAS).

[105] Ok K., Coskun V., Aydin M.N., Ozdenizci B. Current benefits and future directions of NFC services. Proceedings of the 2010 International Conference on Education and Management Technology.

[106] Saraubon K., Chinakul P., Chanpen R. Asset management system using NFC and IoT technologies. Proceedings of the 2019 3rd International Conference on Software and e-Business.

[107] Edwan E., Shaheen F., Shaheen A., Sarsour A. Automated NFC-Based System for Management and Tracking of Assets in Sharing Economy. Proceedings of the 2019 International Conference on Promising Electronic Technologies (ICPET).

[108] Kumar M. (2024). Disadvantages of NFC Business Cards. https://www.hihello.com/blog/disadvantages-of-nfc-business-cards.

[109] Nelson D., Qiao M., Carpenter A. (2013). Security of the near field communication protocol: An overview. J. Comput. Sci. Coll..

[110] Kumar S. (2023). CYBER CRIME: A Review. Int. J. Adv. Sci. Innov..

[111] Moore T., Clayton R., Anderson R. (2009). The economics of online crime. J. Econ. Perspect..

[112] Francis L., Hancke G., Mayes K., Markantonakis K. Potential misuse of NFC enabled mobile phones with embedded security elements as contactless attack platforms. Proceedings of the 2009 International Conference for Internet Technology and Secured Transactions,(ICITST).

[113] Akinyokun N., Teague V. Security and Privacy Implications of NFC-enabled Contactless Payment Systems. Proceedings of the 12th International Conference on Availability, Reliability and Security.

[114] Hartoneva M. (2020). Contactless Payments with Inherence: Strong Customer Authentication And biometrics. https://www.theseus.fi/handle/10024/346627.

[115] Wazid M., Das A.K., Khan M.K., Al-Ghaiheb A.A.D., Kumar N., Vasilakos A.V. (2017). Secure Authentication Scheme for Medicine Anti-Counterfeiting System in IoT Environment. IEEE Internet Things J..

[116] Khalil G., Doss R., Chowdhury M. (2019). A Comparison Survey Study on RFID Based Anti-Counterfeiting Systems. J. Sens. Actuator Netw..

[117] Best M. (2012). Practices.

[118] Levitin A.J. (2010). Private Disordering-Payment Card Fraud Liability Rules. Brooklyn J. Corp. Financ. Commer. Law.

[119] Mann R.J. (2023). Payment Systems and Other Financial Transactions: Cases, Materials, and Problems.

[120] Guo Y., Bao Y., Stuart B.J., Le-Nguyen K. (2018). To sell or not to sell: Exploring sellers’ trust and risk of chargeback fraud in cross-border electronic commerce. Inf. Syst. J..

[121] Scanio S., Glasgow J.W. (2015). Payment card fraud, data breaches, and emerging payment technologies. Fidel. Law J..

[122] Ehabe E. (2023). Attacks on Near Field Communication Devices. https://repository.stcloudstate.edu/cgi/viewcontent.cgi?article=1197&context=msia_etds.

[123] Beck S., Raavi M., Dale C., Weishalla K., Worrell B. Survey of Side-Channel Vulnerabilities for Short-Range Wireless Communication Technologies. Proceedings of the 2024 IEEE International Conference on Electro Information Technology (eIT).

[124] CBNA What Are Phishing and Skimming? Find Out Before Using Your Card. https://cbna.com/blog/financial-wellbeing/what-are-phishing-and-skimming.

[125] Guarascio M., Zuppelli M., Cassavia N., Caviglione L., Manco G. Revealing MageCart-like Threats in Favicons via Artificial Intelligence. Proceedings of the 17th International Conference on Availability, Reliability and Security.

[126] Rus C., Sarmah D.K., El-Hajj M. Defeating MageCart Attacks in a NAISS Way. Proceedings of the 20th International Conference on Security and Cryptography.

[127] Akamai (2024). What Is Magecart? Protecting Your Business from Online Credit Card Skimming. https://www.akamai.com/glossary/what-is-magecart.

[128] Pietro R.D., Oligeri G., Salleras X., Signorini M. N-Guard: A Solution to Secure Access to NFC tags. Proceedings of the 2018 IEEE Conference on Communications and Network Security (CNS).

[129] Raso E., Bianco G.M., Bracciale L., Marrocco G., Occhiuzzi C., Loreti P. (2022). Privacy-aware architectures for NFC and RFID sensors in healthcare applications. Sensors.

[130] Usman A.A., Sani S., Shehu B., Abubakar A. (2024). The Use of Contactless Payment Method to Promote Cashless in Birnin Kebbi Central Market, Kebbi State-Nigeria. Int. J. Res. Publ. Rev..

[131] Akana T., Ke W. (2020). Contactless Payment Cards: Trends and Barriers to Consumer Adoption in the US.

[132] Agrawal S. (2021). Integrating Digital Wallets: Advancements in Contactless Payment Technologies. Int. J. Intell. Autom. Comput..

[133] Straits Research (2024). Wearable Payments Market Size, Share & Trends Analysis Report By Device Type. https://straitsresearch.com/report/wearable-payments-market#:~:text=The%20global%20wearable%20payments%20market,period%20(2024%20%E2%80%93%202032).

[134] rfidspecialist (2023). Which NFC Readers Are Most Commonly Used for The development of Information Solutions with NFC Technology?. https://rfidspecialist.eu/which-nfc-readers-are-most-commonly-used-for-the-development-of-information-solutions-with-nfc-technology--09-10-2023.html.

[135] Ahamad S.S. (2021). A novel NFC-based secure protocol for merchant transactions. IEEE Access.

[136] Thammarat C. (2020). Efficient and Secure NFC Authentication for Mobile Payment Ensuring Fair Exchange Protocol. Symmetry.

[137] Sethia D., Gupta D., Saran H., Agrawal R., Gaur A. (2016). Mutual authentication protocol for secure NFC based mobile healthcard. IADIS Int. J. Comput. Sci. Inf. Syst..

[138] de Carvalho Videira H. (2023). The offline digital currency puzzle solved by a local blockchain. arXiv.

[139] Ibrahim A.O., Ismael Y.H. (2022). EMV Electronic Payment System and its Attacks: A Review. Al-Rafidain J. Comput. Sci. Math. (RJCM).

[140] Chabbi S., Araar C. RFID and NFC authentication protocol for securing a payment transaction. Proceedings of the 2022 4th International Conference on Pattern Analysis and Intelligent Systems (PAIS).

[141] Rayani P.K., Changder S. (2023). Continuous user authentication on smartphone via behavioral biometrics: A survey. Multimed. Tools Appl..

[142] Khan H.U., Sohail M., Nazir S., Hussain T., Shah B., Ali F. (2023). Role of authentication factors in Fin-tech mobile transaction security. J. Big Data.

[143] Alshammari M., Nashwan S. (2022). Fully Authentication Services Scheme for NFC Mobile Payment Systems. Intell. Autom. Soft Comput..

[144] Nicholson T., Hayes D., Le-Khac N.-A. (2023). Forensic Analysis of the iOS Apple Pay Mobile Payment System. IFIP International Conference on Digital Forensics.

[145] Vishwakarma P.P., Tripathy A.K., Vemuru S. (2021). Fraud detection in nfc-enabled mobile payments: A comparative analysis. Innovative Data Communication Technologies and Application: Proceedings of ICIDCA 2020.

[146] Yang M.-H., Luo J.-N., Vijayalakshmi M., Shalinie S.M. (2022). Contactless Credit Cards Payment Fraud Protection by Ambient Authentication. Sensors.

[147] Khalilzadeh J., Ozturk A.B., Bilgihan A. (2017). Security-related factors in extended UTAUT model for NFC based mobile payment in the restaurant industry. Comput. Hum. Behav..

[148] Wang F., Yang N., Shakeel P.M., Saravanan V. (2024). Machine learning for mobile network payment security evaluation system. Trans. Emerg. Telecommun. Technol..

[149] Liebenau J., Elaluf-Calderwood S., Hosein G., Kärrberg P. (2011). Near Field Communications: Privacy, Regulation & Business Models. https://eprints.lse.ac.uk/82485/1/Near%20Field%20Communications%20%5BNFC%5D_%20Privacy%2C%20Regulation%2C%20and%20Business%20Models%20_%20LSE%20Network%20Economy%20Forum.pdf.

[150] Huang R.H., Lam L., Oi A., Yu T., Wing A., Wang C.M. (2021). The Development and Regulation of Mobile Payment: Chinese Experiences and Comparative Perspectives. WashU Glob. Stud. L. Rev..

[151] (2024). NFC-Forum. Industry Body Supports Emerging Regulatory Requirements for Sustainable Product Development. https://nfc-forum.org/news/2024-01-industry-body-supports-emerging-regulatory-requirements-for-sustainable-product-development/#_ftn1.

[152] Sutherland T. (2023). 5 Legal Requirements of Using Contactless Technology in Physical Retail Businesses. https://legalvision.co.uk/regulatory-compliance/contactless-technology-legal-requirements/.

[153] PCI (2019). PCI Contactless Payments on COTS (CPoC™) Standard Provides Security and Test Requirements for Solutions that Enable Contactless Payment Acceptance on Merchant Mobile Devices Using NFC. https://www.pcisecuritystandards.org/about_us/press_releases/pci-security-standards-council-publishes-new-standard-for-contactless-payments/.

[154] (2019). Symantec. Symantec Endpoint Protection Mobile. https://docs.broadcom.com/doc/endpoint-protection-mobile-en.

[155] McAfee (2019). McAfee Complete Data Protection. https://partners.trellix.com/enterprise/en-us/assets/data-sheets/ds-complete-data-protection.pdf.

[156] Povolny S. (2020). The Tradeoff Between Convenience and Security—A Balance for Consumers & Manufacturers. https://www.mcafee.com/blogs/other-blogs/mcafee-labs/the-tradeoff-between-convenience-and-security-a-balancing-act-for-consumers-and-manufacturers/.

[157] Mullick A., Senguptta S. Machine Learning-Based Analysis of IoT Healthcare Data—A Review of Contemporary Research. Proceedings of the 2024 International Conference on Computer, Electrical & Communication Engineering (ICCECE).

[158] Kaspersky (2024). What Is Mobile Security? Benefits, Threats, and Best Practices. https://www.kaspersky.com/resource-center/definitions/what-is-mobile-security.

[159] Kaspersky (2023). Tap-to-Pay, Insert-to-Rob: Cybercriminals Can Now Block Contactless Payments. https://www.kaspersky.com/about/press-releases/tap-to-pay-insert-to-rob-cybercriminals-can-now-block-contactless-payments.

[160] Kaspersky (2023). Using a Token and Smart Card with Authentication Agent. https://support.kaspersky.co.uk/kes-for-windows/11.7.0/133615.

[161] Cisco (2024). Cisco Secure Is the Industry’s Most Complete Open Platform, Securing Your Organization’s Resilience Across Multiple Domains. https://www.cisco.com/c/en/us/buy/enterprise-agreement/security.html.

[162] IBM (2024). Let’s Make Trust the Financial World’s Universal Currency. https://www.ibm.com/blockchain/industries/financial-services.

[163] IBM (2024). IBM Safer Payments: Protect All Cashless Payments from Fraud. https://www.ibm.com/products/safer-payments.

[164] Foresiet (2024). Advanced Android Malware Targets NFC Data for ATM Cashouts. https://foresiet.com/blog/advanced-android-malware-targets-nfc-data-for-atm-cashouts.

[165] NVD (2024). CVE-2024-0568 Detail. https://nvd.nist.gov/vuln/detail/CVE-2024-0568.

[166] NVD (2019). CVE-2019-9295 Detail. https://nvd.nist.gov/vuln/detail/CVE-2019-9295.

[167] NVD (2020). CVE-2020-0022 Detail. https://nvd.nist.gov/vuln/detail/CVE-2020-0022.

[168] NVD (2023). CVE-2023-46765 Detail. https://nvd.nist.gov/vuln/detail/CVE-2023-46765.

[169] NVD (2019). CVE-2019-13943 Detail. https://nvd.nist.gov/vuln/detail/CVE-2019-13943.

[170] NVD (2023). CVE-2023-35671 Detail. https://nvd.nist.gov/vuln/detail/CVE-2023-35671.

[171] Schoon B. Android Loophole Allows Google Wallet to Leak Credit Card Details via NFC, Fix Coming. https://9to5google.com/2023/09/13/android-nfc-credit-card-detail-loophole/.

[172] NVD (2024). CVE-2024-38381 Detail. https://nvd.nist.gov/vuln/detail/CVE-2024-38381.

[173] Hunt R. PKI and digital certification infrastructure. Proceedings of the Ninth IEEE International Conference on Networks, ICON 2001.

[174] NVD (2019). CVE-2019-2114 Detail. https://nvd.nist.gov/vuln/detail/CVE-2019-2114.

[175] NVD (2024). CVE-2024-24313 Detail. https://nvd.nist.gov/vuln/detail/CVE-2024-24313.

[176] Khan A., Glinkin I. (2024). Unveiling Vulnerabilities in Cybersecurity: A Penetration Test Journey. https://www2.deloitte.com/xe/en/pages/about-deloitte/articles/sustainable-strategies/unveiling-vulnerabilities-in-cybersecurity.html.

